# Adverse events analysis as an educational tool to improve patient safety culture in primary care: A randomized trial

**DOI:** 10.1186/1471-2296-12-50

**Published:** 2011-06-14

**Authors:** Clara González-Formoso, María Victoria Martín-Miguel, Ma José Fernández-Domínguez, Antonio Rial, Fernando Isidro Lago-Deibe, Luis Ramil-Hermida, Margarita Pérez-García, Ana Clavería

**Affiliations:** 1 Quality Unit, Vigo Primary Care Region, Galician Health Service, (Rosalía de Castro 21-23), Vigo (36201), Spain; 2 Health Center of Matamá, Vigo Primary Care Region, Galician Health Service, (Babio s/n), Vigo (36312), Spain; 3 Teaching Unit of Family and Community Medicine, Galician Health Service, (Avenida de Zamora 13), Ourense (32005), Spain; 4 Department of Methodology, Faculty of Psychology, University of Santiago de Compostela, (Campus Sur s/n), Santiago de Compostela, (15872), Spain; 5 Health Center of Sárdoma, Galician Health Service, (Baixada a Laxe 76), Vigo, (36204), Spain; 6 Health Center of Fontenla Maristany, Ferrol Primary Care Region, Galician National Health Service. (Plaza de España 19), Ferrol (15403), Spain; 7 Health Center of Mariñamansa, Galician Health Service, (Peña Rey s/n), Ourense, (32005), Spain

## Abstract

**Background:**

Patient safety is a leading item on the policy agenda of both major international health organizations and advanced countries generally. The quantitative description of the phenomena has given rise to intense concern with the issue in institutions and organizations, leading to a number of initiatives and research projects and the promotion of patient safety culture, with training becoming a priority both in Spain and internationally. To date, most studies have been conducted in a hospital setting, even though primary care is the type most commonly used by the public, in our experience.

Our study aims to achieve the following:

- Assess the registry of adverse events as an education tool to improve patient safety culture in the Family and Community Teaching Units of Galicia.

- Find and analyze educational tools to improve patient safety culture in primary care.

- Evaluate the applicability of the Hospital Survey on Patient Safety Culture by the Agency for Healthcare Research and Quality, Spanish version, in the context of primary health care.

**Design and methods:**

**Design**

Experimental unifactorial study of two groups, control and intervention.

**Study population**

Tutors and residents in Family and Community Medicine in last year of studies in Galicia, Spain.

**Sample**

From the population universe through voluntary participation. Twenty-seven tutor-resident units in each group required, randomly assigned.

**Intervention**

Residents and their respective tutor (tutor-resident pair) in teaching units on Family and Community Medicine from throughout Galicia will be invited to participate. Tutor-resident pair that agrees to participate will be sent the Hospital Survey on Patient Safety Culture. Then, tutor-resident pair will be assigned to each group-either intervention or control-through simple random sampling. The intervention group will receive specific training to record the adverse effects found in patients under their care, with subsequent feedback, after receiving instruction on the process. No action will be taken in the control group. After the intervention has ended, the survey will once again be provided to all participants.

**Outcome measures**

Change in safety culture as measured by Hospital Survey on Patient Safety Culture

CONSORT Extension for Non-Pharmacologic Treatments 2008 was applied.

**Discussion:**

The most significant limitations on the project are related to selecting a tool to measure the safety environment, the training calendar of residents in Family and Community Medicine in last year of studies and the no-answer bias inherent to research conducted through self-administered surveys.

The development and application of a safety culture in the health sector, specifically in primary care, is as yet limited. Thus, identifying the strengths and weaknesses in the safety environment may assist in designing strategies for improvement in the primary care health centers of our region.

**Trial registration:**

ISRCTN: ISRCTN41911128

## Background

Present-day health care in developed countries, with its complex combination of technologies, processes and human interactions, has brought great benefits to patients owing to its greater effectiveness. However, this fact, coupled with changing patient characteristics-patients are older, with greater morbidity and require more complex approaches-means that health care entails greater risks and a greater likelihood of causing damage to the patient.

Health care has sought to ensure that diagnostic and therapeutic processes received by patients do not cause them greater harm or injuries than the ailment itself, in an equilibrium that is beneficial to properly managing the process. But even though there has been clear concern for the negative effects of health care, it was not until the appearance of the report by the Institute of Medicine (IOM) *To Err is Human *[1] and the creation by the World Health Organization (WHO) of the World Alliance for Patient Safety [2] that this issue captured the attention of health care providers and political leaders at an international level.

One of the studies on which the IOM report was based was one conducted at Harvard [3] in the 1980's which concluded that nearly 4% of patients suffer some form of harm during a hospital stay, of which 70% suffered temporary damage and 14% resulted in death for the patient. It estimated that between 44,000 and 98,000 people die in hospitals each year as a result of adverse events (AE), figures that exceed mortality rates from traffic accidents, breast cancer or Acquired Immunodeficiency Syndrome (AIDS). The United Kingdom Department of Health, in its annual report of 2000, *An Organisation with a Memory*, estimated that AE occurs in nearly 10% of hospitalizations [4] however, in Australia, the AE rate was 16.6% among patients admitted to a hospital [5]. The cost of these events is quite high, and it is compounded by the erosion in patients' trust, safety and satisfaction.

Since then, a number of studies have been published on the frequency of AE linked to health care, their effect on patients, the potential impact on health systems and the need to study them; most studies have been conducted in a hospital setting [6-9], although experiences are emerging in other environments like primary care (PC) [10-13]. This has led to broader recognition of the problem, the inclusion of safety targets in strategic improvement plans in health organizations and more extensive research into the matter.

In 2006, the Ministry of Health and Social Policy (MSPS) presented its Quality Plan for the Spanish National Health System identifying better safety for patients in the care of the National Health System-see http://www.msps.es/organizacion/sns/planCalidadSNS/home.htm-as a strategy for fostering clinical excellence. Among other initiatives, financing was provided for the research projects "National Study on Adverse Effects Linked to Hospitalization. ENEAS 2005" [14] and "Study on Patient Safety in Primary Health Care. APEAS Study" [15]. The results of the latter study show that primary care practice in Spain is relatively safe, with a prevalence of AE of 18.63%, 70% of which are avoidable, with a predominance of mild cases. It is estimated that AE cases may affect 7 of every 100 people per year.

A cornerstone of the international movement for patient safety and an objective of the MSPS strategy is to foster a safety culture at all levels of health care with a proactive, preventive and educational management approach. Safety culture can be described as the common values, beliefs, behaviours, perceptions and attitudes held by the staff in a health center [16].

The WHO's World Alliance for Patient Safety created a working group with international experts to identify overall priorities in patient safety, with a prominent place for "poor safety culture" in the developed countries [17].

Multiple tools have been developed and evaluated to assess patient safety culture [18-24]. The choice among them will depend on their expected use, the target population, reliability, validity and other considerations [25].

Nearly all these tools sufficiently cover dimensions such as leadership, policies and procedures, professionals and communication and presentation of results [26]. Patient safety culture is a relatively new field [27] and a majority of the studies published on it are based on hospital studies [28]. Few studies have been done on patient safety culture in primary health care [26,29,30] and to date, none in Galicia.

Aside from this information and the existing antecedents, it has become necessary to deal with a crucial component in this entire machinery: medical professionals themselves. Medical professionals are the leading agents providing health care and they are responsible for a great deal of the organization's clinical and social outputs. Their attitudes and perceptions, their degree of involvement and commitment to collective challenges, their working environment and conditions, communication, workload and a huge variety of factors will result in either more or less safety for the patient. Thus, the implementation of a true patient safety culture in primary health services must be one of the main challenges faced in the immediate future, where training will play a key role.

Professionals are needed with knowledge and skills in areas like application of the best available evidence, communication and dialogue. They must perceive that the enhancement of care quality and of factors that help make health care safer are essential in the exercise of the profession.

Quite often, medical students or residents are not seen as an important part of ensuring patient safety. They are an unexploited resource for preventing medical errors, as they should be trained to recognize errors and speak openly about them as a tool for enhancing care quality and learning. Tutors must supervise and encourage students and residents to actively report errors or incidents they observe and prevent the intimidation of the medical hierarchy from frustrating their potential as safety advocates [31]. There are now certification systems in medical education that acknowledge the lack of training in patient safety and have included it among the basic skills [32].

In Spain, post-graduate education is provided through internal resident programs (MIR in Spanish) that must be successfully completed in order to practice the profession. The Family and Community Medicine teaching units (Medicina de Familia in Spanish) are the structures for planning and developing post-graduate education in the field. The present curriculum in the field, which was approved in 2004 [33], provides innovation as an educational assessment strategy with the development of the so-called Thoughtful Practice Guide, which includes a series of creative learning tools [34-36].

These tools are diverse and applicable to several educational areas: one of these is the self-audit [37] of clinical histories to investigate whether a certain care practice to be measured meets pre-defined and desired standards of quality. It is aimed at finding practical solutions to deficiencies. The very act of reflecting upon the results obtained and expected is educational; its educational potential has been recognized because it helps medical professionals become aware that their clinical practice is not perfect and that they need to improve on a continuous basis; it acts as a powerful motivational incentive by serving as a method of self-modifying conduct; it enables the identification of educational needs; it helps to improve the efficiency of clinical practice by detecting practices that are unnecessary or inconsistent with the professional's own standards, and contributes to improving the effectiveness of the health care provided to patients.

On the basis of the experience gained through participating in the APEAS, recording AE and the AE studies in primary care pediatrics of the Vigo health care area and in the Tuberculosis Unit, we hypothesize that action consisting of awareness-raising, recording, notification and handling of AE in medical own consultation is a formative assessment tool that meets the needs of adult learning by improving and promoting patient safety culture among participating professionals.

Given the need identified by the WHO and the MSPS for research into methods of learning and fostering patient safety culture among health organizations and professionals, we believe it is pertinent, timely and justified to undertake this research project into tools for enhancing patient safety culture in primary care by promoting awareness-raising and learning. Thus, we pose two hypotheses to be tested:

1. The registry of adverse events is a teaching tool that has a positive impact on patient safety culture in the Family and Community Medicine teaching units of Galicia.

2. The Hospital Survey on Patient Safety Culture (SOPS) is valid for use in primary health care services.

## Methods/Design

### Design overview

**Figure 1:**
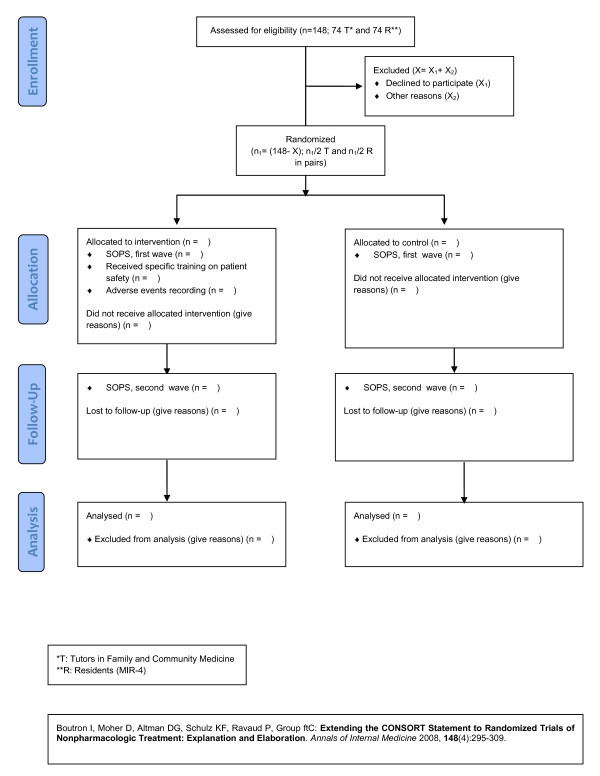
Summary of trial design

For the first hypothesis: Educational intervention study. Experimental unifactorial design of two groups-control and intervention-with pre-and post-test recording of the patient safety culture in Family Medicine teaching units with the SOPS (Additional file 1), as per residents' answers. In the intervention group, tutor training on patient safety will also be conducted, followed by the recording of adverse effects with the APEAS survey and feedback (Figure 1).

For the second hypothesis: Psychometric study of SOPS, including exploratory factor analysis, internal consistency and item analysis.

### Study setting and population

#### Setting

The Spanish National Health System is a system of universal coverage (including illegal immigrants). Services are free at the point of delivery, with the exception of prescription drugs to persons under 65 years of age, who should participate in paying with a contribution of 40% of retail, with some exceptions. The health powers are transferred entirely to the regions since late 2002. This decentralization resulted in 17 health departments (ministries or departments of health), the most common structure of regional health systems comprising a health department, responsible for regulation and policy planning, and a regional health service which is responsible for providing services. The territorial organization of health services is based on health areas; the primary care network is public and most of the providers are employees of public sector professionals. Primary care centers are staffed by a multidisciplinary team of family physicians, pediatricians, nurses and social workers, some also have physiotherapists, midwives, pharmacists, dentists and hygienists; they are assigned basic laboratory and diagnostic imaging, which can be found in the center or be centralized and serve several establishments.

Health expenditure reached U.S. $ 2,671 per capita (in PPP terms) and 8.5% of GDP in 2007, 71% is paid by public funds (collected mainly through taxes), which is intended to 16% primary care. The autonomous communities manage 90% of public health resources. In Galicia, with a population of 2,700,000, there are 7 areas of health, 398 primary care health centers and 3141 general practitioners, each of which serves on average 1500 citizens. Each of them has a training unit of Family and Community Medicine.

#### Population

Tutors belonging to the seven Family and Community Medicine teaching units of Galicia and residents in the last year of studies (MIR-4) of Family and Community Medicine.

In 2009, there were 160 tutors and 288 residents, of which 69 are MIR-4. As these residents and their tutors constitute the so-called "tutor-resident pair", we will request the participation of 35 tutors and 35 residents in the intervention group and 34 tutors and 34 residents in the control group.

### Measurements

#### Adverse events

The APEAS form (Additional file 2) was elaborated from the form created by the Medical School of the University of Washington in its project on patient safety and adapted after the results of the ENEAS study [14] with consensus techniques [15]. It consists of 11 modules that analyze the following: risk factors for the patient, a summary of the event and its possible cause, the care level at which it took place, its impact on the patient, the effects caused in the patient, the health care received by the patient as a result of the AE, the causal factors of the AE, the extent to which health care was the cause of the lesion, the evidence that the AE could have been avoided, an assessment of the evidence of possible prevention and what could have been done to prevent the problem. Although the study was conducted with the collaboration of PC professionals throughout the country without seeking a significant sample, it is of a seminal nature in Spain.

#### Culture

The SOPS consists of 44 questions and measures 14 dimensions. It was developed by Westat under contract with the Agency for Healthcare Research and Quality (AHRQ), and 16 of the questions were derived from a revision of existing instruments. The questionnaire was piloted in 20 hospitals, and the results were used to generate a list of 14 factors, all of which showed a high degree of internal consistency in factorial analysis (0.63 to 0.84). It emphasizes the institutional commitment to safety, transfers and transitions and teamwork. A summary of the technique and issues related to the development of the instrument and other tools is available http://www.ahrq.gov/qual/patientsafetyculture/.

### Intervention and randomization

#### Intervention

Intervention consists of three different moments, which will occur in this order:

##### a) Training workshops

Each participant is to be provided with current data on the incidence of adverse events in primary care and they will be acquainted with current initiatives in both Spain and internationally.

Program:

Theory: Introduction to patient safety; concepts of adverse effect, incident, adverse event, complication, secondary effect, adverse drug reaction; types of errors and their analysis; errors related to the use of drugs.

Practice: Manual recording of AE in the notification survey. The functioning of the form used in the APEAS will be presented and explained [15].

Duration

Each workshop will last approximately 2 hours.

Teacher team

The teacher team will be composed of two people, the same in the 8 workshops, one family doctor and a nurse.

Place and dates of workshops

One workshop will be conducted in each of the 7 health areas included, except in Lugo, where 2 will be given owing to the geographic dispersion of the learning centers, on their premises and with a timetable adapted to the centers' activity.

##### b) Recording adverse events with the APEAS form

Professionals in the tutor-resident pairs in the intervention group will manually complete, voluntarily and anonymously, the APEAS form whenever they identify an adverse effect in their offices during the recording period (15 days) in the course of their daily activities.

##### c) Feedback

Every participant will send the field coordinator the AE recorded along with data on daily activity in the forms. The field coordinator will, in turn, return to medical professionals the analysis of their records at the end of the study.

#### Randomization

Every tutor-resident pair in Galicia will be invited to participate. Tutor-resident pairs will be assigned randomly to experimental groups (control vs. intervention) through use of the SPSS 17 statistical package, by one team researcher not belonging to Primary Care. The pair will be assigned randomly to groups by stratifying the teaching unit to ensure their equivalence in relation to a number of variables of interest and to avoid possible underlying biases. Special care will be taken that both the intervention group and the control group will be comprised of the same number of individuals and that their distribution by health centers and areas will be also similar.

Twenty-four tutor-resident units in each group are required to detect an improvement in patient safety culture of 30% with 80% power and confidence level of 95%. If we estimate 10% in losses, 27 tutor-resident units per group must be captured.

Although the sample size calculated was 27, it was decided to invite the total population for three reasons: 1) Being an educational intervention, by the very execution of the survey (SOPS), and a research project at the same time. 2) In each of the 7 teaching units, a pair-resident tutor would be excluded, a priori; while statistically defensible, it would not be so from an organizational perspective, working environment, and the like. 3) Exactly match the sample size for efficiency and ethics of the study is not applicable in this case.

### Measurement of results

For the first hypothesis, the dependent variable will be patient safety culture as measured by the SOPS. The independent or explanatory variable will be participation in the intervention-training workshops, recording of adverse events in daily work and reception of feedback.

For the second hypothesis, the following will be analyzed: reliability (internal consistency, repeatability), validity (content, criteria, construct) and change sensitivity (size of effect, paired t).

### Data analysis

For the first hypothesis:

• Descriptive data analysis with traditional univariate character tabulation with central trend measurement and variability.

• Comparisons between groups and phases, with use of parametric bivariate contrasts (t of Student and simple variance analysis) and non-parametric ones (Wilcoxon and Kruskal Wallis).

• To measure the possible effect of the intervention and possible interaction, a variance analysis of repeated measurements will be used.

For the second hypothesis:

• Analysis of scale psychometric properties. To study the dimensionality or factor structure, an exploratory factorial analysis will be made, using the principal components method. To analyze reliability, indicators will be collected for internal consistency (Cronbach's α) and to study the criteria or predictive validity, an analysis of correlations and a multiple linear regression analysis will be made.

Finally, a descriptive analysis of declared adverse events will be undertaken

All analyses will be made with the SPSS 17 statistical package. Statistical significance will be assigned to p values lower than 0.05.

### Ethical approval and informed consent

This research has been authorized by the Ethical Committee of Clinical Research of Galicia and informed consent will be solicited from participants in the study, wherein the information sheet will explain the voluntary nature of collaborating and the participant's freedom to change procedures without giving any explanation. Thus, the confidentiality of both the participation and the information collected is ensured by its anonymous handling.

The study will observe basic ethical principles and the provisions of the law at all times.

### Phases of study

A working calendar of some 12 months was established for performing the study as such, followed by data analysis and dissemination of results.

In Phase 1 (first quarter), the main objective was to publicize the project and arouse interest so as to enable us to achieve a high degree of participation. Those interested were provided with detailed information on the process and the conditions.

The Family and Community Medicine teaching units throughout Galicia were contacted to invite tutors and residents to participate in the study. Participation was promoted by a drawing for a PDA and laptop computer among participants.

To ensure that participation in the study proceeds within a formal framework, each participant was sent a letter from the lead researcher in the project, along with an informed consent letter to be signed. A second letter was to be sent by the coordinator of field work, providing more specific details on the process and the phases of the study.

Based on the first version of the survey, the first phase included a review that will involve the participation of both experts and physicians, which might ensure that the wording of the questions was suitable. At the same time, all the materials necessary were prepared for the execution of the following phases.

The tutor-resident pair was assigned to each group-intervention or control-by simple random sample.

In Phase 2 (second quarter), a first use was made of the SOPS. This phase must was completed by both the control and intervention groups.

In Phase 3 (third quarter), an intervention was made in one of the groups pursuant to the protocol set forth above. In the control group, no action was taken.

In Phase 4 (fourth quarter), participants in both the control and intervention groups were again be provided with the SOPS to be filled out. The time between the two uses of the questionnaire was 4 months, an interval considered suitable, as it is too large for individuals to remember the answers given in the first phase and, at the same time, sufficient for the effect of the intervention to have been instilled and materialized in the daily work of medical professionals.

Phase 5 (third semester) was consist of a review of all the questionnaires, their encoding and recording in computer format and a statistical analysis of the data.

Finally, Phase 6 (fourth semester), at present, is the dissemination phase, with production of publications and presentation of results in the participating health centers.

## Discussion

In our analysis of this research, we have encountered certain difficulties that were evaluated in a realistic manner before initiating execution of the project.

Although a number of tools have been developed to assess organizational culture [38], these tend to measure a large number of dimensions without focusing specifically on patient safety [39]. However, surveys centered on patient safety culture have appeared [40], as have reviews of these surveys [23].

The psychometric properties and dimensionality of the AHRQ questionnaire have recently been reviewed by Sorra and Dyer [39] with a survey among 331 hospitals, 2267 hospital services and 50513 survey respondents. The results support the 12 dimensions and 42 themes included in the SOPS, with psychometric properties acceptable at all levels of analysis, with few exceptions.

Although other instruments are available for measuring the safety environment, the one elaborated by the AHRQ was chosen owing to its rigorous process of construction and validation [41], with psychometric properties confirmed in the version adapted in Spanish (Analysis of Patient Safety Culture in Hospitals of the Spanish National Health System [*Análisis de la cultura sobre seguridad del paciente en el ámbito hospitalario del Sistema Nacional de Salud español*] [42] and with extensive experience of application in the United States [43].

It is possible that SOPS, even if it is initially oriented to both hospital and non-hospital environments and culturally validated for a Spanish setting, may not faithfully reflect the characteristics of primary care in our region. Thus, difficulties may arise in the reading and interpretation of items and dimensions. Nevertheless, adaptation to this care level is an important step forwards in patient safety culture, which is less developed and studied at this care level.

APEAS questionnaire was proposed for the registration of adverse events, being the only state-wide used in Spain, allowing us to compare the results. It does not correspond to the taxonomy currently proposed by WHO [44-46], that was later published.

Specialized training in family and community medicine in Spain is conducted through the MIR system (MIR is Spanish for resident internal doctor), and access to it is by means of a competitive exam and a standard that measures candidates' academic transcripts and ranks them by scores composed of the sum of the exam grade and the transcript grades, whereby candidates can select their field of study as per this order. For every specialized field-including, of course, family medicine-there is a national commission that establishes the curriculum of study, the MIR requirements, certification of educational institutions and units, quality control of the structure, process and results, among others.

The most recent family medicine curriculum in Spain was approved in 2005. It is a highly ambitious, quite complete and detailed program that sets out each objective according to an order of priority within each defined skill area. Training is distributed among the 4 years planned in rotations through different hospitals and the health center for the initial 6 months and the last year of residence. In family medicine, unlike other hospital specializations, each resident has an assigned tutor in the health center work alongside them through their residence and supervise the fulfilment of objectives as part of a learning evaluation that identifies possibilities for improvement and proposes corrective measures, with constant feedback in continued active tutoring meetings.

One of the significant advances and strengths of this pioneering program is the implementation of learning evaluation through the completion of a reflective practice guide, in which residents think about the tools being used and the tutors supervise the method.

As per the training calendar of family MIR, this study should closely match the established timetable, as residents in family medicine will be in the health center for 1 year before completing their training, and then they will disperse. Thus, the performance period is limited by the difficulty caused by the need to re-establish contact with residents once their training is over. This demands significant discipline and dedication, as a multicenter study with substantial distances between participating centers makes coordination particularly arduous, a factor to be taken into account when considering the obstacles to be overcome.

The inherent characteristics of primary care in Spain, where the accessibility of care is foremost, exert special pressure on medical professionals, as they have little control over the distribution of daily work, with the subsequent conflicts of space and time for the inclusion of research work. Therefore, this difficulty in managing and organizing work constitutes a major barrier to the execution of any kind of research and, in the best of cases, proves to be a special burden no matter how small the tasks may be.

## List Of Abbreviations

AE**: **Adverse Events; AHRQ**: **Agency for Healthcare Research and Quality; AIDS**: **Acquired Immunodeficiency Syndrome; APEAS**: **Study on Patient Safety in Primary Health Care; ENEAS**: **National Study on Adverse Effects Linked to Hospitalization; GDP**: **Gross Domestic Product; IOM**: **Institute of Medicine; MIR**-**4**: **Training doctors in the 4th year of family medicine; MSPS**: **Ministry of Public Health and Social Affairs; PC**: **Primary care; PPP**: **Purchasing Power Parity; SOPS**: **Hospital Survey on Patient Safety Culture; WHO**: **World Health Organization

## Definitions

Adverse event

Set of incidents and adverse effects.

Adverse effect

Any unforeseen and unexpected accident identified upon medical examination that has caused an injury and/or incapacity due to health care received and not the patient's base ailment. To determine whether the adverse event is due to care, reviewers score on a scale of 6 points (1 = no evidence or little evidence; 6 = practically certain evidence) their degree of confidence that the AE is due to health care and not the pathological process. A priori, we would use a cut-off point of ≥ 2 to consider it to be positive.

Avoidable adverse effect

To determine whether the adverse event is avoidable, reviewers will score on a scale of 6 points (1 = no evidence or little evidence; 6 = practically certain evidence) their degree of confidence that the adverse effect is avoidable. A cut-off point of ≥ 4 is used to consider it to be positive. Determinations: AE frequency. Proportion of avoidable AEs.

Grave adverse effect

Causing death, residual incapacity upon release or requiring surgery.

Moderate adverse effect

Causes hospital stay of at least 1 day or requires emergency or specialist care.

Mild adverse effect

Injury or complication that causes none of the above.

Incident

Unforeseen and unexpected random event that causes no harm to the patient. It can also be defined as an event that in different circumstances might have been an adverse event or an event leading to problems for the patient if not discovered or corrected in time.

Medical error

Mistake or omission in the practice of medical professionals that may contribute to the occurrence of an adverse event.

Drug error

Effect that can be avoided and which is caused by the improper use of a medication, causing harm to a patient while under the care of a medical professional, patient or consumer.

Adverse drug reaction

Alteration and/or lesion caused when medications are used appropriately.

## Competing interests

The authors declare that they have no competing interests.

## Authors' contributions

CGF conceived of the study, participated in its design, coordinated the research team, gave training workshops and worked on centralizing the data, and draft the manuscript. VMM conceived of the study, participated in its design and helped to draft the manuscript. MaJFD participated in its design and in the coordination of the teaching unit of Orense and helped to draft the manuscript. ARB performed the statistical analysis and helped to draft the manuscript. FLD participated in its design, coordinated work in the Vigo and Pontevedra teaching units. LRH participated in the design of the study and coordinated work in the teaching units of A Coruña, Santiago and Lugo. MPG participated in the design of the study and in the coordination of the UD of Orense and in the coordination of the UD of Orense All authors read and approved the final manuscript. AC participated in its design and helped to draft the manuscript.

## Authors' information

CGF received a Diploma of Advanced Studies for the project "Adverse Events in Primary Pediatric Care." She conducts research into issues related to patient safety as a member of the Citizen Network of Trainers in Patient Safety in the Spanish Ministry of Health and Consumer Affairs.

VMM, Public health technician in the management of primary care in Vigo and in the Family and Community Medicine teaching unit of Vigo, she is helping to draft the Master Evaluation Plan of Family and Community Medicine Teaching Units of Galicia and of a software application for the development of the plan; also, since 2005, she has been a research fellow in the Galicia, Association for Research of Family and Community Medicine. She is a member of the Primary Care Research Network in Vigo and Pontevedra (INAPPOVI group) funded by the Government of Galicia's Directorate General of Research, Development and Innovation.

M^a^JFD is a health technician in the management of primary care in Orense, working on research projects and coordinating the training program in family and community medicine of the Orense province. At present, she is part of the health care quality task force of the Orense government engaged in six primary care improvement projects, one of which is "Preventing Drug-Related Adverse Effects."

ARB. Professor of the Department of Methodology of Behavioral Sciences. University of Santiago de Compostela. Expert in health care quality and marketing. Author of more than 50 publications in international journals. 15 years of teaching experience from different masters courses and doctoral programs. Project Evaluator, National Evaluation and Foresight (ANEP) of the Ministry of Education and Culture. Principal Investigator in more than a hundred research projects and technical reports. Director of 8 PhD theses.

FLD Tutor in the MIR training program of Family and Community Medicine in the teaching unit of Vigo from 7 June 1988 until 2 Nov. 2005 and coordinator of the teaching unit of Vigo since 1 Feb. '05. Since 2007, tutors research work in the inter-university doctoral program (Universities of Vigo and Santiago de Compostela).

MPG is a tutor in the Family and Community Medicine unit of Orense. Member of the task force on hypertension for the Galician Association of Family and Community Medicine.

AC is a specialist in preventive medicine, having taken part in a number of research projects in primary care; also a member of the INAPPOVI group, which is recognized by the Government of Galicia's Directorate General of Research, Development and Innovation; co-directs two theses that began as projects funded in a public call by the Health Research Fund (FIS in Spanish) of the Ministry of Health and Social Affairs. Owing to her professional work, she has extensive experience in coordinating projects for managing change in health organizations in both primary care and hospitals, in addition to administrative positions in the Department of Health of the Government of Galicia and the Spanish Ministry of Health and Consumer Affairs. At this time, she is working on a project financed by the FIS under code: PI10/01172. Project Evaluator, National Evaluation and Foresight (ANEP) of the Ministry of Education and Culture. She has participated in the design of the electronic medical history of primary care in Galicia, and has published in prominent journals.

## Pre-publication history

The pre-publication history for this paper can be accessed here:

http://www.biomedcentral.com/1471-2296/12/50/prepub

## Supplementary Material

Additional file 1 http://www.biomedcentral.com/imedia/1502762492556180/supp1.doc. Click here for file

Additional file 2 http://www.biomedcentral.com/imedia/2898093725561793/supp2.doc. Click here for file
