# Supplementary material for: Adverse events analysis as an educational tool to improve patient safety culture in primary care: A randomized trial
Source: BMC Fam Pract. 2011 Jun 14;12:50. doi: 10.1186/1471-2296-12-50 (PMC3142500; doi:10.1186/1471-2296-12-50)
Supplement: Additional file 1 — http://www.biomedcentral.com/imedia/1502762492556180/supp1.doc. [file 1471-2296-12-50-S1.DOC]

**Appendix 1: SOPS** (English version)


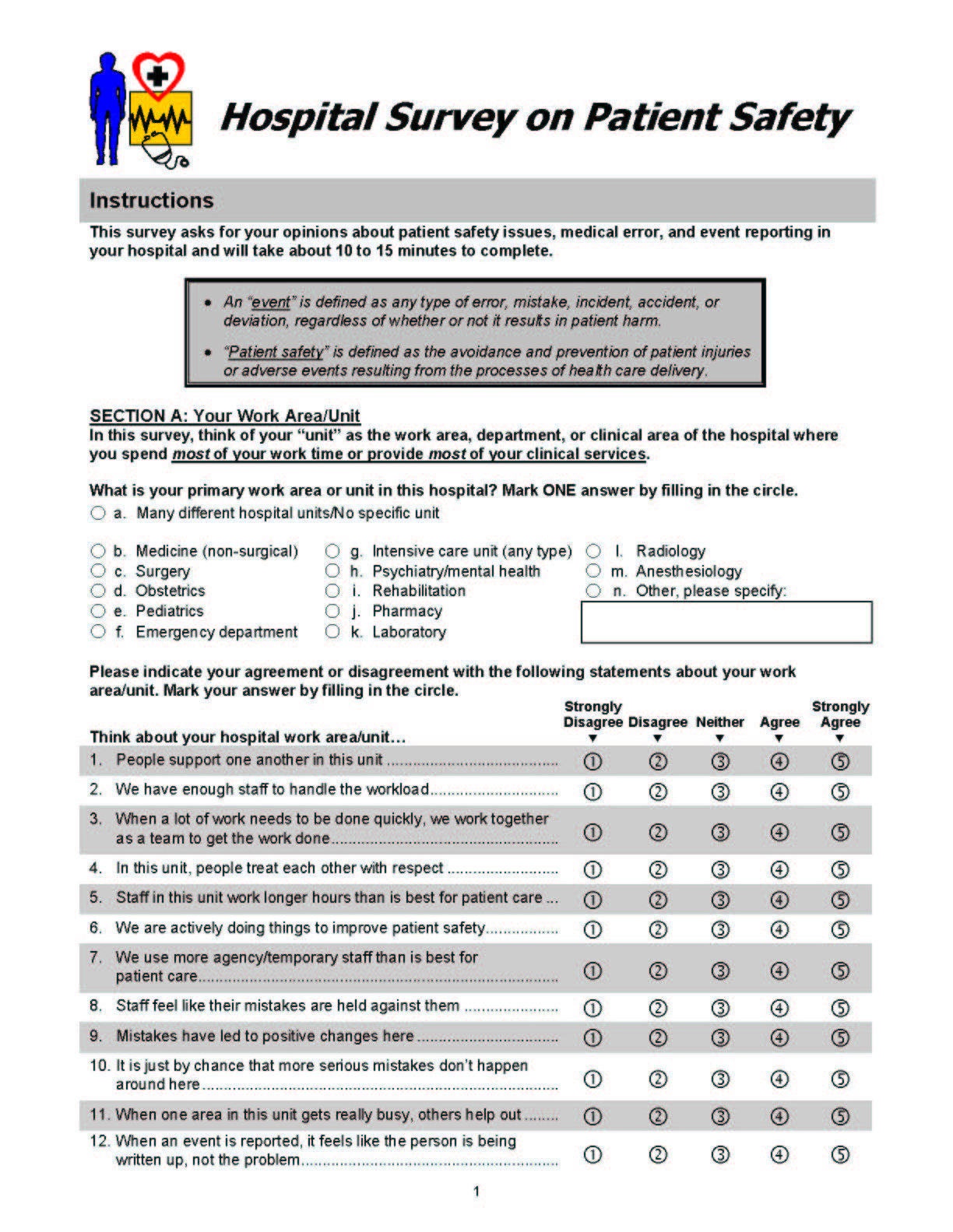


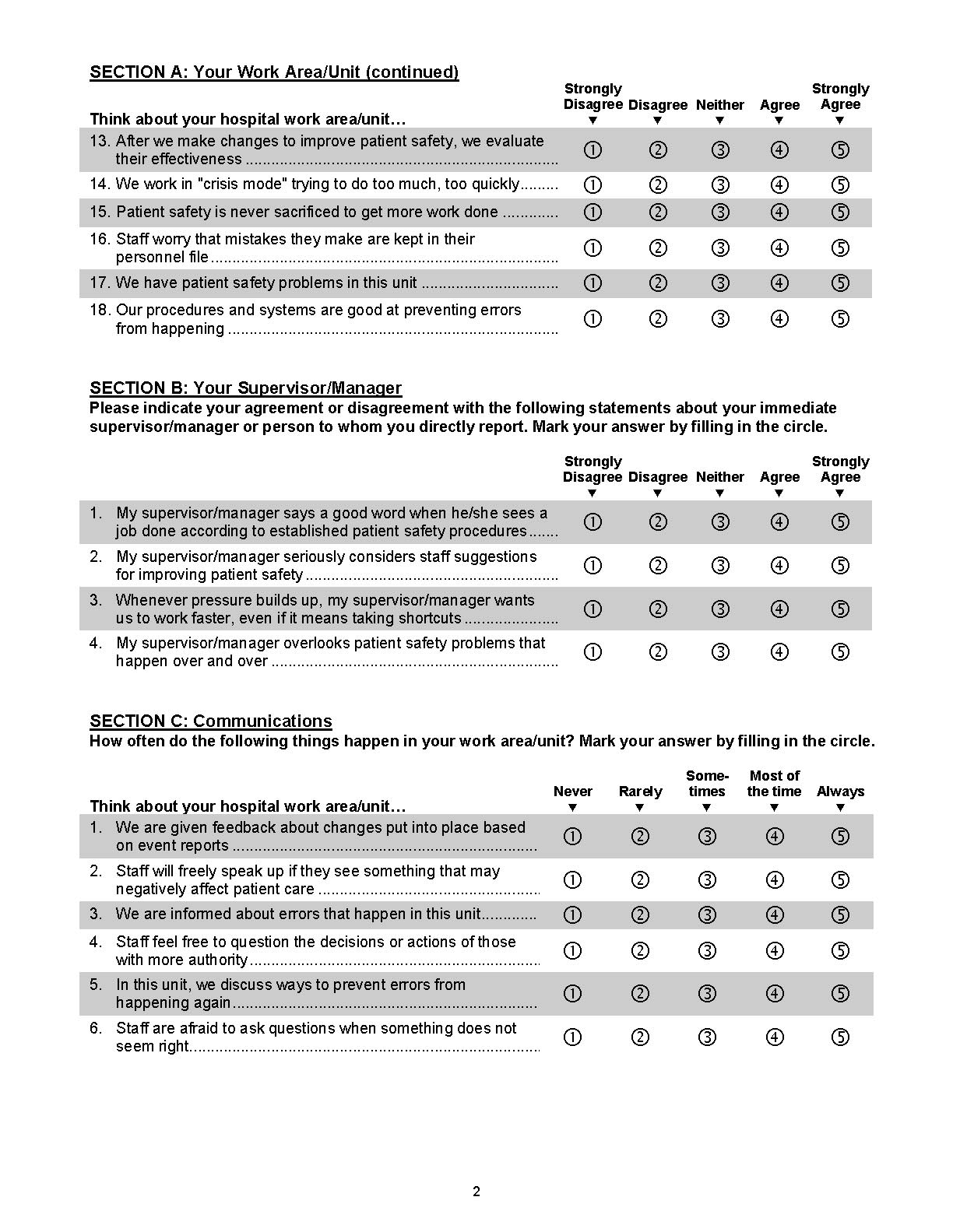


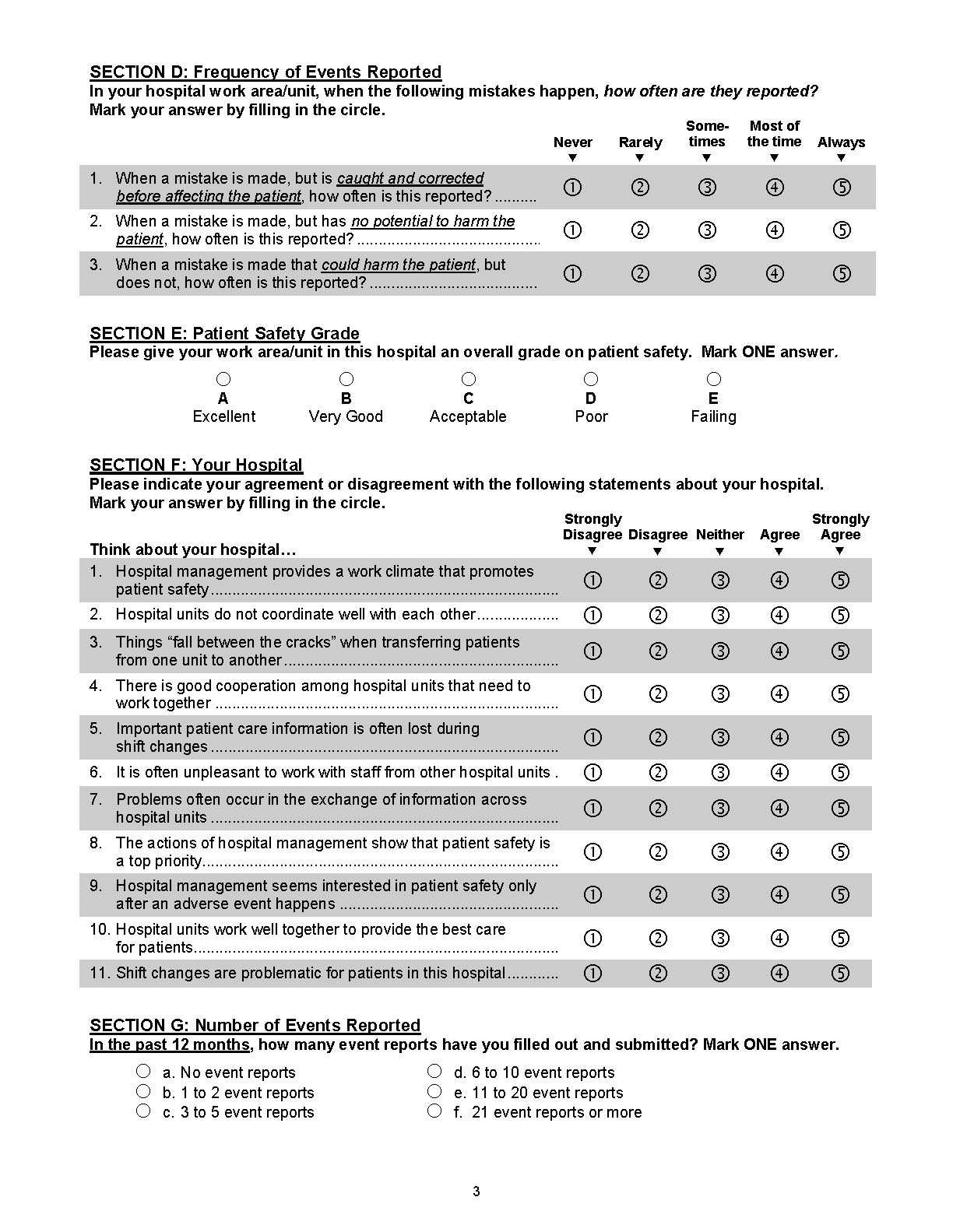


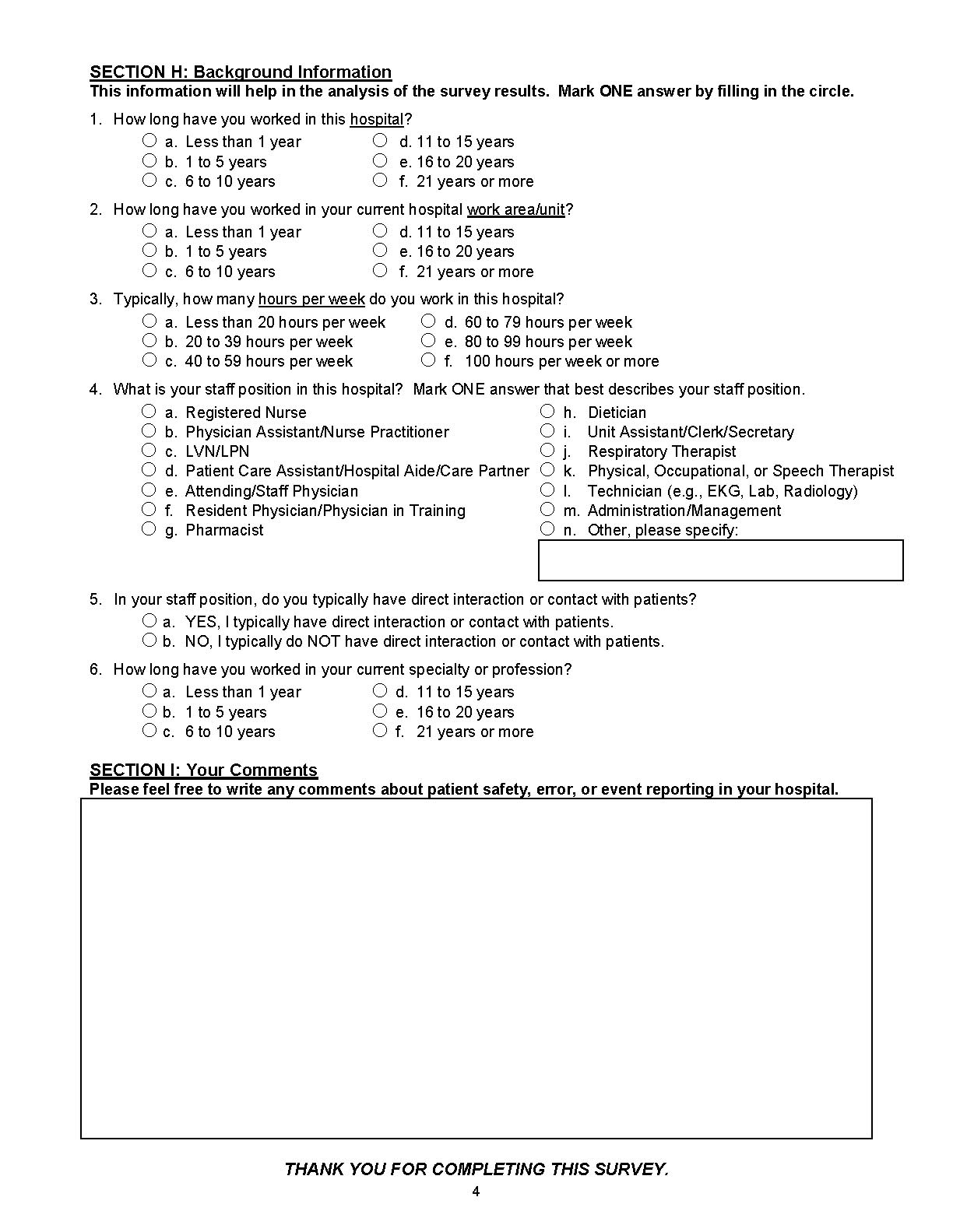


**Appendix 1: SOPS (Spanish version)**


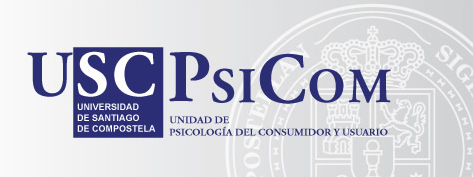

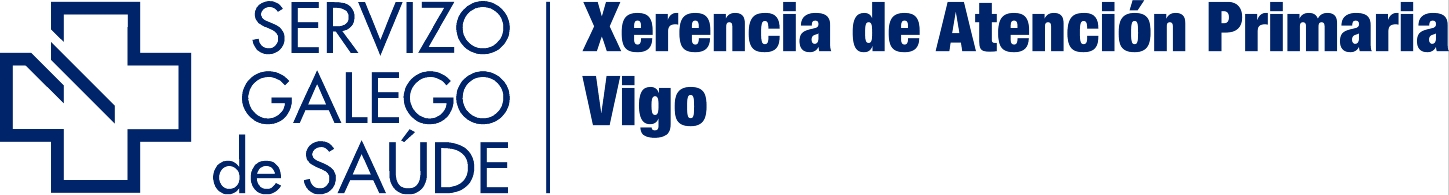

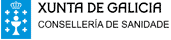


**Fecha de Cumplimentación**: ____ / ____ /____

A través de las Unidades Docentes de Galicia estamos realizando un estudio para evaluar la cultura de la seguridad del paciente de nuestros profesionales. Para ello utilizamos un cuestionario validado por la OMS y la AHRQ (*Agency for Healthcare Research and Quality*), que es necesario que cumplimentes con atención y sinceridad. Es importante que tengas en cuenta que no hay respuestas “correctas” ni “incorrectas”. Lo único que se pretende es conocer cómo está siendo el funcionamiento del centro en el que trabajas, con relación a algunos de los temas objeto de estudio, así como tus propias opiniones al respecto.

Se trata de un cuestionario muy sencillo que, por lo general, se cumplimenta en 10-15 minutos. No obstante, si tienes cualquier duda o hay alguna cuestión que te resulte confusa, no dudes en ponerte en contacto con nosotros para que podamos aclararla.

Queremos insistir también en que todas tus respuestas van a ser tratadas de manera absolutamente confidencial, por lo que te rogaríamos que intentases responder a todas ellas. No obstante, en caso de que alguna pregunta no se refiera al trabajo que desempeñas, puedes dejarla en blanco.

**DEFINICIONES PREVIAS:**

- Un **“suceso”** es definido como cualquier tipo de error, equivocación, incidente, efecto adverso o actuación fuera de protocolo, sin importar si el resultado daña al paciente o no.
- **“Seguridad del paciente”** se define como el evitar y prevenir lesiones en los pacientes o efectos adversos como resultado de los procesos de cuidados de salud prestados.
- **“Unidad”:** el área de trabajo, servicio, departamento o área clínica del centro donde usted pasa la mayor parte de su horario de trabajo o proporciona sus servicios clínicos.

**¿Cuál es su principal área o UNIDAD de trabajo? Seleccione SÓLO UNA respuesta.**

Ámbito hospitalario

- Diversas unidades del centro/Ninguna unidad específica
- Medicina (No-quirúrgica)
- Cirugía
- Obstetricia
- Pediatría
- Área de Urgencias
- Unidad de cuidados intensivos (general, coronaria, pediátrica…)
- Salud mental/Psiquiatría
- Rehabilitación
- Farmacia
- Laboratorio
- Radiología
- Anestesiología
- Otros, por favor, especifique:

Ámbito Atención Primaria

- Medicina de Familia
- Pediatría de Familia
- Enfermería Comunitaria
- Otros, por favor especifique:

**Sección A: Área/Unidad de Trabajo**

**Piense en su servicio, unidad o área de trabajo… ¿En qué medida está Ud. de acuerdo con cada una de las siguientes afirmaciones?**

|  | **Muy en desacuerdo** | **En desacuerdo** | **Ni de acuerdo ni en**  **desacuerdo** | **De acuerdo** | **Muy de acuerdo** |
| --- | --- | --- | --- | --- | --- |
| 1. El personal de esta unidad suele apoyarse mutuamente | 🞏1 | 🞏2 | 🞏3 | 🞏  4 | 🞏5 |
| 1. Tenemos suficiente personal para afrontar la carga de trabajo habitual | 🞏1 | 🞏2 | 🞏3 | 🞏  4 | 🞏5 |
| 1. Cuando tenemos mucha carga de trabajo, trabajamos en equipo para terminarlo | 🞏1 | 🞏2 | 🞏3 | 🞏  4 | 🞏5 |
| 1. En esta unidad el personal se trata con respeto | 🞏1 | 🞏2 | 🞏3 | 🞏  4 | 🞏5 |
| 1. El personal de esta unidad trabaja más horas de lo que sería conveniente para una buena atención al paciente | 🞏1 | 🞏2 | 🞏3 | 🞏  4 | 🞏5 |
| 1. Tenemos actividades específicas dirigidas a mejorar la seguridad del paciente | 🞏1 | 🞏2 | 🞏3 | 🞏  4 | 🞏5 |
| 1. Tenemos más personal de sustitución o eventual del que es conveniente para una buena atención al paciente | 🞏1 | 🞏2 | 🞏3 | 🞏  4 | 🞏5 |
| 1. El personal siente que sus errores son utilizados en su contra | 🞏1 | 🞏2 | 🞏3 | 🞏  4 | 🞏5 |
| 1. Cuando se detecta algún fallo se ponen en marcha medidas para evitar que vuelva a ocurrir | 🞏1 | 🞏2 | 🞏3 | 🞏  4 | 🞏5 |
| 1. En esta unidad no se produce un mayor número de errores por pura casualidad | 🞏1 | 🞏2 | 🞏3 | 🞏  4 | 🞏5 |
| 1. Cuando alguien está sobrecargado de trabajo, suele encontrar ayuda en sus compañeros | 🞏1 | 🞏2 | 🞏3 | 🞏  4 | 🞏5 |
| 1. Cuando se informa de un efecto adverso, parece que se está juzgando a la persona más que al problema en sí mismo | 🞏1 | 🞏2 | 🞏3 | 🞏  4 | 🞏5 |
| 1. Después de introducir cambios para mejorar la seguridad de los pacientes, solemos evaluar su efectividad | 🞏1 | 🞏2 | 🞏3 | 🞏  4 | 🞏5 |
| 1. Frecuentemente trabajamos bajo presión, intentando hacer muchas cosas y en muy poco tiempo | 🞏1 | 🞏2 | 🞏3 | 🞏  4 | 🞏5 |
| 1. La seguridad del paciente nunca se compromete por hacer más trabajo | 🞏1 | 🞏2 | 🞏3 | 🞏  4 | 🞏5 |
| 1. El personal de la unidad teme que los errores que puedan cometer consten en su expediente | 🞏1 | 🞏2 | 🞏3 | 🞏  4 | 🞏5 |
| 1. En esta unidad tenemos problemas con la seguridad de los pacientes | 🞏1 | 🞏2 | 🞏3 | 🞏  4 | 🞏5 |
| 1. Nuestros procedimientos y sistemas son efectivos para la prevención de errores | 🞏1 | 🞏2 | 🞏3 | 🞏  4 | 🞏5 |

**Sección B: Jefe/Supervisor**

**Piense en su superior/jefe inmediato o persona de la que usted depende directamente. ¿En qué medida está Ud. de acuerdo con cada una de las siguientes afirmaciones?**

|  | **Muy en desacuerdo** | **En desacuerdo** | **Ni de acuerdo ni en**  **desacuerdo** | **De acuerdo** | **Muy de acuerdo** |
| --- | --- | --- | --- | --- | --- |
| 1. Mi superior/jefe hace comentarios favorables cuando ve un trabajo hecho de conformidad con los procedimientos establecidos | 🞏1 | 🞏2 | 🞏  3 | 🞏  4 | 🞏5 |
| 1. Mi superior/jefe considera seriamente las sugerencias del personal para mejorar la seguridad de los pacientes | 🞏1 | 🞏2 | 🞏  3 | 🞏  4 | 🞏5 |
| 1. Cuando la presión de trabajo aumenta, mi superior   /jefe quiere que trabajemos más rápido, aunque se ponga en riesgo la seguridad de los pacientes | 🞏1 | 🞏2 | 🞏  3 | 🞏  4 | 🞏5 |
| 1. Mi superior/jefe no hace caso de los problemas de seguridad de los pacientes aunque se repitan una y otra vez | 🞏1 | 🞏2 | 🞏  3 | 🞏  4 | 🞏5 |

**Sección C: Comunicación**

**¿Con qué frecuencia se plantean las siguientes cuestiones en su servicio/unidad/área de trabajo?**

|  | **Muy en desacuerdo** | **En desacuerdo** | **Ni de acuerdo ni en**  **desacuerdo** | **De acuerdo** | **Muy de acuerdo** |
| --- | --- | --- | --- | --- | --- |
| 1. Se nos informa sobre los cambios realizados a partir de los sucesos que hemos notificado | 🞏1 | 🞏2 | 🞏3 | 🞏  4 | 🞏5 |
| 1. El personal comenta libremente si ve algo que podría afectar negativamente al cuidado del paciente | 🞏1 | 🞏2 | 🞏3 | 🞏  44 | 🞏5 |
| 1. Se nos informa sobre los errores que se cometen en esta unidad | 🞏1 | 🞏2 | 🞏3 | 🞏  4 | 🞏5 |
| 1. El personal se siente libre de cuestionar las decisiones   o acciones de aquellos con mayor autoridad | 🞏1 | 🞏2 | 🞏3 | 🞏  4 | 🞏5 |
| 1. En esta unidad discutimos cómo se pueden prevenir los errores para que no vuelvan a suceder | 🞏1 | 🞏2 | 🞏3 | 🞏  4 | 🞏5 |
| 1. El personal tiene miedo de hacer preguntas cuando algo aparentemente no está bien | 🞏1 | 🞏2 | 🞏3 | 🞏  4 | 🞏5 |

**Sección D: Frecuencia de Sucesos Notificados**

**En su servicio/unidad/área de trabajo, ¿con qué frecuencia son notificados, cuando ocurren los siguientes errores?**

|  | **Muy en desacuerdo** | **En desacuerdo** | **Ni de acuerdo ni en**  **desacuerdo** | **De acuerdo** | **Muy de acuerdo** |
| --- | --- | --- | --- | --- | --- |
| 1. Cuando se comete un error, pero es descubierto y corregido antes de afectar al paciente, ¿con qué frecuencia es notificado? | 🞏1 | 🞏2 | 🞏3 | 🞏  4 | 🞏5 |
| 1. Cuando se comete un error, pero no tiene el potencial de dañar al paciente, ¿con qué frecuencia es notificado? | 🞏1 | 🞏2 | 🞏3 | 🞏  44 | 🞏5 |
| 1. Cuando se comete un error que pudiese dañar al paciente, pero no lo hace, ¿con qué frecuencia es notificado? | 🞏1 | 🞏2 | 🞏3 | 🞏  4 | 🞏5 |

**Sección E: Valoración de la Seguridad del Paciente**

**A continuación debe realizar una valoración general de su servicio, unidad o área de trabajo, en lo que a la seguridad del paciente se refiere.**

| **Excelente** | **Muy Bueno** | **Aceptable** | **Pobre** | **Malo** |
| --- | --- | --- | --- | --- |
| 🞏 | 🞏 | 🞏 | 🞏 | 🞏 |

**Sección F: Su Centro**

**Por favor, indique su acuerdo o desacuerdo con las siguientes afirmaciones sobre su centro.**

|  | **Muy en desacuerdo** | **En desacuerdo** | **Ni de acuerdo ni en**  **desacuerdo** | **De acuerdo** | **Muy de acuerdo** |
| --- | --- | --- | --- | --- | --- |
| 1. La Dirección de este centro propicia un ambiente laboral que promueve la seguridad del paciente | 🞏  1 | 🞏2 | 🞏3 | 🞏  4 | 🞏5 |
| 1. Los servicios o unidades de este centro no se coordinan bien entre ellos | 🞏  1 | 🞏2 | 🞏3 | 🞏  4 | 🞏5 |
| 1. La información de los pacientes se pierde, en parte, cuando son trasladados de un servicio o unidad a otro | 🞏  1 | 🞏2 | 🞏3 | 🞏  4 | 🞏5 |
| 1. Hay buena cooperación entre los servicios o unidades del centro que necesitan trabajar juntos | 🞏  1 | 🞏2 | 🞏3 | 🞏  4 | 🞏5 |
| 1. A menudo se pierde información importante de los pacientes durante los cambios de turno | 🞏  1 | 🞏2 | 🞏3 | 🞏  4 | 🞏5 |
| 1. En este centro, con frecuencia resulta incómodo trabajar con personal de otros servicios o unidades | 🞏  1 | 🞏2 | 🞏3 | 🞏  4 | 🞏5 |
| 1. Con frecuencia es problemático el intercambio de información entre los servicios o unidades del centro | 🞏  1 | 🞏2 | 🞏3 | 🞏  4 | 🞏5 |
| 1. Las acciones de la Dirección del centro muestran que la seguridad del paciente es altamente prioritaria | 🞏  1 | 🞏2 | 🞏3 | 🞏  4 | 🞏5 |
| 1. La Dirección del centro sólo parece interesada en la seguridad del paciente después de que ocurre un suceso | 🞏  1 | 🞏2 | 🞏3 | 🞏  4 | 🞏5 |
| 1. Los servicios o unidades del centro trabajan juntos y de forma coordinada para dar la mejor atención a los pacientes | 🞏  1 | 🞏2 | 🞏3 | 🞏  4 | 🞏5 |
| 1. Los cambios de turno son problemáticos para los pacientes que acuden al centro | 🞏  1 | 🞏2 | 🞏3 | 🞏  4 | 🞏5 |

**Sección G: Número de Sucesos Notificados**

**1. ¿Existe en su centro un procedimiento para notificar incidentes o efectos adversos?**

🞏 Si 🞏 No

**2. En los pasados 12 meses, ¿cuántas veces ha notificado por escrito un incidente o efecto adverso a su superior o a otras instancias?**

- Ninguna notificación 🞏 De 6 a 10 notificaciones
- De 1 a 2 notificaciones 🞏 De 11 a 20 notificaciones
- De 3 a 5 notificaciones 🞏 21 notificaciones o más

**Sección H: Perfil de los Participantes**

1. **¿Cuánto tiempo lleva usted trabajando en este centro?**

- Menos de 1 año 🞏 De 11 a 15 años 🞏 MIR
- De 1 a 5 años 🞏 De 16 a 20 años
- De 6 a 10 años 🞏 21 años o más

**2. ¿Cuánto tiempo lleva usted trabajando en su actual especialidad?**

- Menos de 1 año 🞏 De 11 a 15 años 🞏 MIR
- De 1 a 5 años 🞏 De 16 a 20 años
- De 6 a 10 años 🞏 21 años o más

**3. Habitualmente, ¿cuántas horas a la semana trabaja usted en este centro?**

- Menos de 20 horas a la semana 🞏 De 40 a 59 horas a la semana
- De 20 a 39 horas a la semana 🞏 60 horas a la semana o más

**4. En su puesto de trabajo ¿tiene interacción directa o contacto con los pacientes?**

🞏 SÍ 🞏 NO

**5. ¿Podría decirnos su Edad?**

**Sección I: Comentarios y Sugerencias**

Para terminar, le agradeceríamos que nos hiciera llegar cualquier tipo de comentario, observación o sugerencia que le parezca de interés.

**MUCHAS GRACIAS POR SU COLABORACIÓN**
