# Supplementary material for: Adverse events analysis as an educational tool to improve patient safety culture in primary care: A randomized trial
Source: BMC Fam Pract. 2011 Jun 14;12:50. doi: 10.1186/1471-2296-12-50 (PMC3142500; doi:10.1186/1471-2296-12-50)
Supplement: Additional file 2 — http://www.biomedcentral.com/imedia/2898093725561793/supp2.doc. [file 1471-2296-12-50-S2.DOC]

**Appendix 2: APEAS form (English version)**

**USCPSI.com Government of Galicia Galicia Health Service Administration of Primary Care Vigo**

***Form for detection of AE in primary care***

Center details Patient details

Center type Case __________________

- Urban
- Rural Age___________________

Professional category Professional experience Event date______________

- Family doctor – Less than 1 year
- MIR – Between 1 and 5 years

– Between 6 and 10 years

– More than 10 years

Notification date________________

**1. Indicate whether the patient has any of the following risk factors:**

*INTRINSIC RF*

- Renal insufficiency
- Diabetes
- Neoplasia
- Immunodeficiency
- Chronic pulmonary ailment
- Neutropenia
- Hepatic cirrhosis
- Drug addiction
- Obesity
- Hypoalbuminemia
- Pressure ulcers
- Malformations
- Cardiac insufficiency
- Coronary illness
- Hypertension
- Alcoholism
- Hypercholesterolemia
- Depression
- HIV
- Malaria

*EXTRINSIC RF*

- Open urinary probe
- Closed urinary probe
- Enteral nutrition
- Tracheotomy
- Immunosuppressive therapy
- Colostomy
- Nasogastric probe

**2. Summarize what happened and what you believe the cause was:**

**3. At what level of care did the problem occur?**

- Primary emergency care
- Primary medical care
- Primary nursing care
- Hospital emergency room
- Prior hospital admittance
- Specialized care
- Pharmacy
- Others (physiotherapy, herbalist, etc)

**4. Which of the following statements best describes the impact on the patient?**

- The effect did not occur, but it was about to occur.
- The incident occurred, but it was detected before it could affect the patient.
- The incident occurred and affected the patient, but the patient suffered no harm.
- The effect occurred and the patient suffered temporary harm
- The effect occurred and the patient was in critical condition (e.g., cardiac failure).
- The effect occurred and the patient suffered permanent harm
- The effect occurred and resulted in the death of the patient.

**5. Specify all the effects that occurred in the patient:**

**Related to a procedure**

Hemorrhage or hematoma related to surgery or surgical procedure

- Hematuria related to probe
- Circulatory disorder (very tight ferula)
- Dehiscence of sutures
- Serosa, abscesses or granulomas
- Tympanic perforation
- Other complications owing to surgical procedure

**Related to nosocomial infections**

- Infection of surgical or traumatic wound.
- ITU related to probe
- Bacteremia related to device
- Opportunistic infection from immunosuppressive treatment or antibiotics
- Infection from pressure ulcer
- Aspiration pneumonia

**Care related**

- Phlebitis
- Pressure ulcer
- Burns, abrasions, falls and contusions (including resulting fractures)
- Cystic lesions from injections
- Other consequences of care

**Drug related**

- Nausea, vomiting or diarrhea secondary to medication
- Feeling unwell or pain from drugs
- Pruritus, rash or dermal lesions in reaction to drugs or dressing
- Systematic allergic reactions.
- Cephalgea (headaches) from drugs
- Neurological alterations from drugs
- Constipation
- Other secondary effects (dyspnea, coughing, dry mouth)
- Hypertension from drugs
- Poor control of arterial pressure
- High digestive hemorrhage.
- Hemorrhage from anti-coagulation
- Acute myocardial infarction, stroke, pulmonary embolism, deep venous thrombosis
- Electrolyte imbalance
- Edemas, cardiac insufficiency and shock.
- Alteration of heart rhythm or electrical activity from drugs
- Functional alteration (renal, hepatic, thyroid, etc)
- Poor control of glucose levels
- Neutropenia
- Local effects or fever after vaccination or drug
- Poor pain management

**General**

- Worse evolution of base illness
- Need to repeat procedure or visit
- Anxiety, stress or depression

**Others**

- Other consequence________________
- **No effect**

1. **What care did the patient receive as a result of the adverse effect?**

- Health care was not affected
- Required higher level of observation or PC monitoring
- Required further tests (radiography, analysis) in PC
- Additional medical treatment or surgery (antibiotics or minor surgery in PC)
- Required another visit or referral to specialized care or emergency room without admittance
- Required hospitalization: vital support systems (orotracheal intubation, CPR, surgery).

1. **Indicate all the factors causing the adverse effect**

**Medication-related**

Adverse Drug Reaction

**■** Wrong medicine

**■** Wrong dose

**■** Omission of dose, medication or inoculation

**■** Incorrect frequency of administration

**■** Error in preparation for handling

**■** Insufficient monitoring

**■** Dispensation error

**■** Wrong patient

**■** Incorrect treatment duration

**■** Lack of adherence to treatment

**■** Medication interaction

**Management- related**

**■** Duplicated Clinical History

**■** Error in medical information

(Results from tests of other patients)

**■** Error in the identification of the patient

**■** Prolonged waiting list

**■** Problems with computerized history

**■** Appointment error

**Diagnosis-related**

**■** Diagnosis error

**■** Delay in transfer to specialized attention

**■** Delay in the diagnosis

**Care-related**

**■** Inadequate handling of the patient

**■** Inadequate handling of alert signs

**■** Inadequate handling of technique

**Other**

Other causes _____

1. **To what degree was medical care the cause of the lesion?**

**■** Absence of evidence that the problem was due to patient care. The lesion was owed entirely to the patient’s pathology.

**■** Minimum probability that care was the cause.

**■** Limited probability that care was the cause.

**■** Moderate probability that care was the cause.

**■** It is very probable that care was the cause.

**■** Total evidence that care was the cause of the problem/adverse event.

1. **In your view, is there any evidence that the AE could have been avoided?** __Yes __ No
2. **Rate on a 6-point scale the evidence of the possibility of prevention**
   1. **■** Absence of evidence of the possibility of prevention
   2. **■** Remote possibility of prevention
   3. **■** Some possibility of prevention
   4. **■** Moderate possibility of prevention
   5. **■** Elevated possibility of prevention
   6. **■** Total evidence of the possibility of prevention
3. **What could have been done to avoid this problem?**

**Appendix 2: APEAS form (Spanish version)**
